# The Effect of General Anaesthesia on Circadian Rhythms in Behaviour and Clock Gene Expression of *Drosophila melanogaster*

**DOI:** 10.3390/clockssleep2040032

**Published:** 2020-10-23

**Authors:** Nina Li, Ralf Stanewsky, Tessa Popay, Guy Warman, James Cheeseman

**Affiliations:** 1 Department of Anaesthesiology, School of Medicine, University of Auckland, 1142 Auckland, New Zealand; dongni.li@auckland.ac.nz (N.L.); g.warman@auckland.ac.nz (G.W.); 2 Institute of Neuro-and Behavioral Biology, Westfälische Wilhelms University, 48149 Münster, Germany; stanewsky@uni-muenster.de; 3 Department of Cell and Developmental Biology, Vanderbilt University, Nashville, TN 37240, USA; tessa.m.popay@vanderbilt.edu

**Keywords:** general anaesthesia, circadian clock, *Drosophila*, locomotor activity, phase response curve, *period* gene expression

## Abstract

General anaesthesia (GA) is implicated as a cause of postoperative sleep disruption and fatigue with part of the disturbance being attributed to a shift of the circadian clock. In this study, *Drosophila melanogaster* was used as a model to determine how Isoflurane affects the circadian clock at the behavioural and molecular levels. We measured the response of the clock at both of these levels caused by different durations and different concentrations of Isoflurane at circadian time 4 (CT4). Once characterized, we held the duration and concentration constants (at 2% in air for 6 h) and calculated the phase responses over the entire circadian cycle in both activity and period expression. Phase advances in behaviour were observed during the subjective day, whereas phase delays were associated with subjective night time GA interventions. The corresponding pattern of gene expression preceded the behavioural pattern by approximately four hours. We discuss the implications of this effect for clinical and research practice.

## 1. Introduction

General anaesthesia (GA) provides hypnosis, analgesia, immobility, paralysis, and amnesia depending on concentration and is a prerequisite for surgery. Despite being routinely used for over 150 years, the mechanisms underlying the mode of action of GA are still not completely understood. Previous studies have reported that GA effects many molecular targets [1]. There have been a number of longitudinal studies on the nervous system [2] that have reported the involvement of the Mg^2+^ and Ca^2+^ ion channels [3,4], neurotransmitter receptors [1], and circadian clock genes [5]. In addition, it has been described that ion channels can be regulated by the suprachiasmatic nuclei (SCN), the central circadian clock in mammals, and they also revealed that the influx and efflux of ions can activate or inactivate cellular signalling resulting in changes in circadian clock gene expression [6]. However, the connection and the corresponding relationship between GA and clock gene expression in vivo is poorly understood.

The molecular mechanism of the circadian clock is well conserved across a variety of different taxa [7,8]. In fruit flies, the positive feedback loop is regulated by the heterodimeric transcription factors CLK-CYC (CLK-BMAL1 in mammals), which induce transcription of the negative elements of the loop [9]. The negative feedback loop is regulated by PER-TIM (PER-CRY in mammals) that enter the nucleus and directly interact with CLK-CYC, where they repress their own transcription [2,10]. This inhibition is reversed by proteasome-mediated degradation of the negative elements, initiating a new transcription cycle [8,11].

The circadian clock is synchronised by environmental cues in a process called entrainment [12]. Light is the predominant *Zeitgeber* (time giver) for entrainment, and the phase of the circadian rhythm can be delayed or advanced by a single timed pulse [12]. The magnitude and direction of the phase shift is determined by the application time of the stimulus [13].

We have previously shown in honeybees (*Apis mellifera*), which rely on a time-compensated sun compass, that a six-hour daytime anaesthetic treatment using Isoflurane shifts their flight direction by 60 degrees, and their foraging time is delayed by approximately 3 h 20 min [5]. This behavioural change was accompanied by a similar phase delay in the underlying clock gene *Cry-m* and *per* mRNA expression, which were delayed by 4.9 h and 4.3 h, respectively, in individual bee brains. Interestingly, this delay occurred only following the day-time GA and not during the night. This led us to consider that anaesthesia’s effect on the clock is time–dependent. It is difficult to test this in the honeybee model, as we relied on pooling of individuals for the qPCR and had to sacrifice bees at each time point for serial sampling. In this study, we have taken the advantages of *D. melanogaster* as an established behavioural and genetic model to investigate the effects of GA with Isoflurane on locomotor activity and circadian clock gene expression in vivo.

There were three aims: First, to characterise the effect of varying concentration (dose) and length of anaesthesia on the clock. This has not previously been reported and on the basis that volatile anaesthetic agents act at similar concentrations on a variety of taxa, we chose to test a range of concentrations and duration that might reasonably be considered clinically relevant. Secondly, we constructed a phase response curve to investigate the effect of timing of a standard anaesthetic on the behaviour. Finally, we constructed a similar PRC to determine the effect of timing of anaesthesia on clock gene expression using flies expressing a *period-luciferase* reporter gene.

## 2. Results

### 2.1. Isoflurane Causes Behavioural Phase Shifts in Locomotor Activity

**Figure 1:**
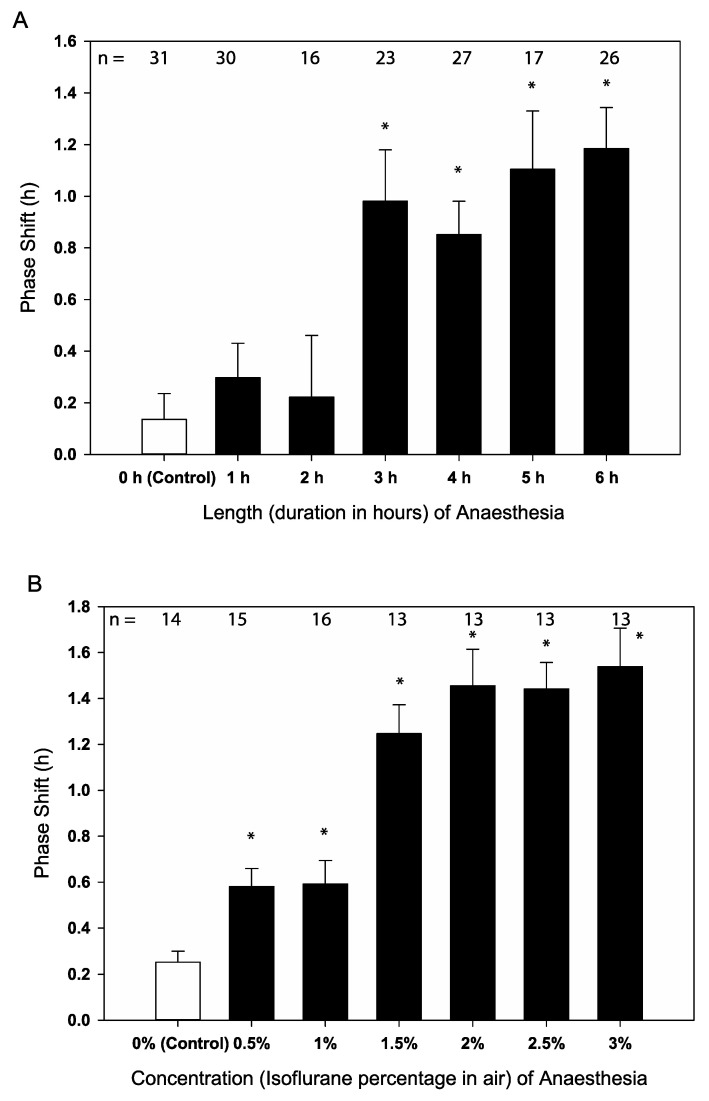
(**A**) The effect of anaesthetic duration on phase (2% Isoflurane treatment centred at circadian time 4 (CT4)). (**B**) The effect of anaesthetic concentration (6 h anaesthesia centred at CT4). Data shown as mean with SEM error bars. Asterisks denote significant difference from the control (Students *t*-test, * *p* < 0.002) shown uncorrected for multiple comparisons. See also Table S1.

Increasing the duration of anaesthesia incrementally at a fixed concentration (2% isoflurane in air) at the same time of day (CT4) caused modest phase advances (between 0.85 and 1.18 h, Students *t*-test *p* < 0.002) in behaviour after three hours (see Figure 1A and Table S1). Anaesthesia of one- or two-hour duration at a fixed concentration and administered at this time showed no significant difference to the controls, which received the same handling without anaesthesia (Students *t*-test *p* > 0.36 and *p* > 0.71).

When the time of day of administration and the duration of anaesthesia were held constant (CT4 and 6 h respectively), increasing the concentration of GA increased the magnitude of the phase shifts from 0.58 h at 0.5% to 1.54 h at 3% concentration (See Figure 1B and Table S2). This pattern is not linear and appears to plateau after concentration exceeds 2% isoflurane in the air suggesting the effect of the concentration of the anaesthetic reaches a ceiling after which no further phase shift can take place at that particular CT.

### 2.2. Behavioural Phase Response to Anaesthesia at Different Times of Day

**Figure 2:**
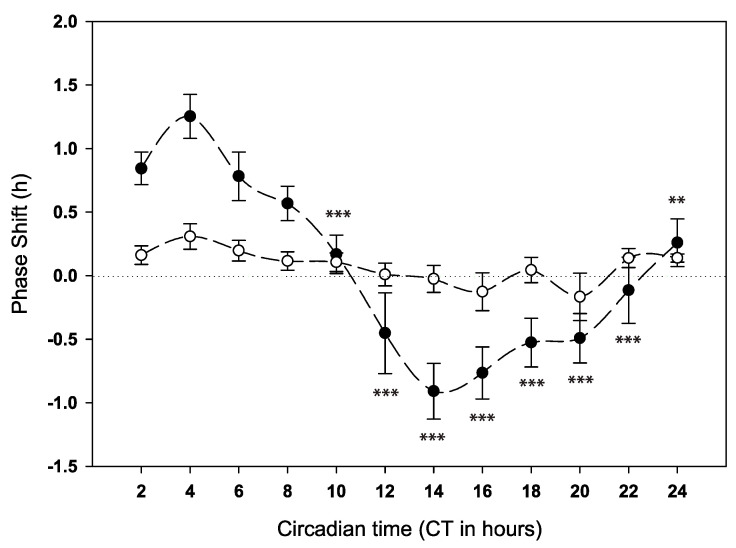
Behavioural phase response curve (PRC) for anaesthesia (6 h of 2% Isoflurane, centred around the respective CT) administered over the circadian cycle in wild-type *D. melanogaster*. Solid circles indicate the mean phase shift to GA, and open circles indicate the associated control. Error bars indicate standard error of the mean. Significant differences compared to the CT4 bin calculated by general linear model (GLM) analysis indicated by asterisks, ** *p* < 0.01, *** *p* < 0.001. See also Table S5 and Figure S1.

To ascertain the phase response, we held the concentration and duration constants (2% at 6 h) and systematically varied the time of day of administration in order to construct the phase response curve. Anaesthesia was given on day five of constant darkness (DD) at each circadian time (CT) to different cohorts of flies each paired with a similar cohort of controls, which received the same handling but without anaesthesia (see PRC Figure 2, Tables S3 and S5, and Actograms in Figure S1). During most of the subjective day, anaesthesia generally caused phase advances in activity (from CT0 to CT8) peaking between CT2 and CT4 (+1.25 h). Phase delays were observed between CT12 and CT22 with the maximum phase delay (~1 h) at CT14. No significant phase changes were observed at CT10 and at the end of the subjective night (CT22 and CT24).

### 2.3. General Anaesthesia Affects Molecular Clock Components in a Time–Dependent Manner

Having characterized the effect of anaesthesia on behaviour, we tested the effect on clock gene expression using the luciferase reporter strain 8.0-luc, which reports *per* expression in a subset of dorsal clock neurons. As in the behavioural experiments, GA was administered for 6 h at 2% concentration at successive times throughout the subjective day and night.

**Figure 3:**
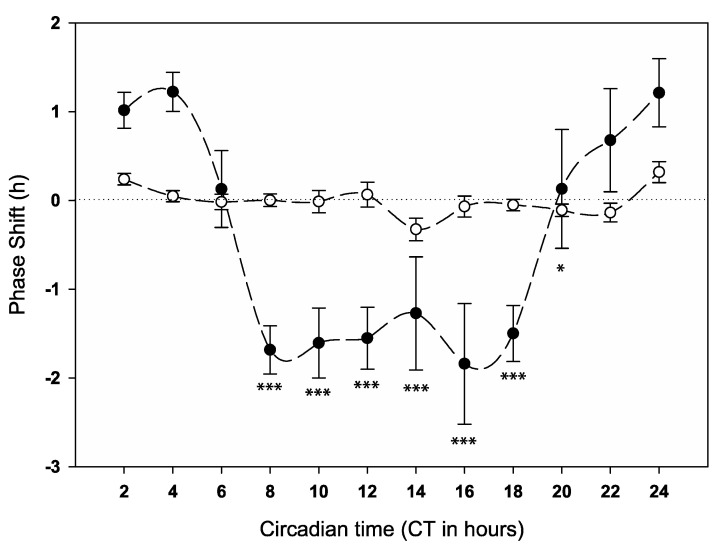
Phase response curve of *per* expression to anaesthesia (6 h of 2% Isoflurane) administered over the circadian cycle in 8.0-luc transgenic D. melanogaster. Solid circles indicate the mean phase shift to GA, and open circles indicate the associated control. Error bars indicate standard error of the mean. Significant differences compared to the CT4 bin calculated by GLM analysis indicated by asterisks, * *p* < 0.05, *** *p* < 0.001. See also Table S6 and Figure S2.

The phase responses of the patterns in bioluminescence (reporting *per* expression) to anaesthesia at different circadian times are plotted in Figure 3 (see also Tables S4 and S6 and Actograms Figure S1). During the late subjective night and early subjective day, anaesthesia causes phase advances in *per* expression from CT0 to CT4 and CT22 to CT24, reaching the highest phase advance of 1.22 h at CT4. From CT8 through to CT18, phase delays were measured with the largest phase delay at CT16 (1.84 h delay). No phase shifts were observed at the middle of the subjective day (CT6) and at CT20.

The pattern of phase shifts in *per* expression followed a similar overall pattern to the behaviour (compare Figure 2 and Figure 3), but the transition from advances to delays occurred approximately four hours earlier than the behavioural shifts. These results support the idea that gene expression precedes, and therefore most likely is causative of, behavioural phase shifts.

## 3. Discussion

The aim of this study was to investigate the effects of anaesthesia on the locomotor behaviour and clock gene expression in *Drosophila*, including defining the effects of the different lengths, concentrations, and times of administration of anaesthesia. Here, for the first time, we have explored in more detail the relationship between concentration and duration of anaesthetic on the clock. At the same circadian phase (CT4) higher concentrations (≥2%), and longer anaesthesia (≥3 h) caused larger phase shifts but this was not a linear effect.

In previous work studying anaesthesia’s effect on the clock in the honeybee our experiments were all based on an anaesthetic concentration of 2% isoflurane for six hours. The rationale for this was two-fold: First in the overall scheme we are interested in anaesthesia’s effects on patients and therefore we chose a “clinically relevant” concentration of 2% which is an effective dose for a variety of animals and humans. Secondly, a six hour duration anaesthetic, should it have “stopped” the clock, would be equivalent to a 90 degree shift in the time-compensated-sun-compass-orientation angle of the bees [5]. The current study suggests that, at least in *Drosophila*, the clock shifting effect of a discrete anaesthetic can occur at lower concentration and after a shorter anaesthetic period. The implication for this is that even with moderate doses, less than required clinically for surgery, anaesthesia can affect the molecular clockwork. However, the results also suggest the effect of anaesthesia on the clock does not increase significantly once a threshold of concentration (>1.5%) or duration (>3 h) is reached. This is of potential clinical interest as 1.5% isoflurane represents a minimum alveolar concentration (MAC) of 1.15 in humans [14] and would be routinely achieved as maintenance doses of isoflurane without neuromuscular blockade are regularly around 2% or more [15].

When the length and concentration of the anaesthetic were kept constant (2% for 6 h) and the circadian time of administration varied, we observed a clear pattern in advances and delays in locomotor activity. The general pattern of the phase response curve was characterized by phase advances early in the subjective morning and day followed by delays after CT10.

Taken together the results of the current locomotor activity experiments in *Drosophila* were consistent, and the results were repeatable. The duration, concentration, and PRC experiments all had a point of intersection where data were collected at CT4 for 6 h at 2% concentration, and these groups all produced similar results (compare Figure 1A,B and Figure 2 and data in Tables S1–S3).

The pattern of anaesthesia’s effect on clock gene expression (*per*) was consistent with the locomotor PRC but advanced by approximately four hours. Interestingly, the magnitude of the delays observed between CT8 and CT18 was greater than that observed in the behaviour. We infer that the internal biological timing process has been reset or disturbed during the GA treatment by affecting at least one of the interlocked feedback loops (PER/TIM), potentially by causing premature degradation of these clock components.

Although collected under different conditions, the results obtained in our *Drosophila* experiments are not entirely consistent with results obtained previously in the honeybee. In contrast to the current *Drosophila* PRCs, we have only single night vs. day data points for the honeybee. Honeybee data were collected in constant dim light conditions (LL_dim_). The effects of GA in the honeybee were delayed in whole-hive-activity rhythms at CT5 and clock gene expression (*per* mRNA and *cry* mRNA) after day-time GA at CT8–10. No shift in whole-hive-activity rhythms (CT 20) or clock gene expression at (CT20–22) were recorded as a result of night-time GA [5]. These points in the honeybee do not fit conveniently on the current *Drosophila* PRCs. It is not immediately clear why this should be, as both species, in the wild at least, are diurnal [16]. However, apart from differences in experimental design the honeybee clock mechanism is different and more mammalian-like than *Drosophila* [17], which leads us to the possibility that the differences in anaesthesia’s effect on the clock may be brought about by different targets in the mechanism.

In the study of circadian rhythms, the phase response curve is often used to decipher the effect of the zeitgeber (usually light or temperature) on the clock and its implications for steady state entrainment. This was not our intention here as the idea of steady state entrainment to anaesthesia is of course not sensible. Furthermore, to describe the maximum phase advances and delays as “the limits of entrainment” is illogical in this context. Rather, we intended to work out the time of day effect of discrete single pulses of the anaesthesia on the clock in the context of what might reasonably be expected for patients undergoing surgery and anaesthesia in clinical practice. The results in *Drosophila* certainly indicate that anaesthesia’s time of day effects change over the circadian cycle and with duration and concentration, and that the associated sleep disturbance, and post-operative recovery as a result, will vary. The obvious next step is to determine whether the components and processes of the clock mechanism are being targeted by anaesthesia, which will rely on experiments in both invertebrate and mammalian models.

## 4. Materials and Methods

Young male adult flies (5–10 days after eclosion) were employed for all behavioural and gene expression experiments. All flies were reared in 12:12 LD cycles controlled by the ClockLab^®^, maintained at 25 °C on a substrate of Formula 4–24 instant *Drosophila* medium.

### 4.1. Locomotor Activity Experiments Including Dose, Duration, and Phase Responses

The locomotor activity of wild-type *Drosophila* was measured using the TriKinetics^®^ DAM System, which infers activity from counts of the number of interruptions of infrared light beams across a series of glass tubes each containing a single fly. In each iteration of the experiment, locomotor activity was recorded in two identical environmental cabinets operating on opposing light and dark cycles for 13 days. The animals were entrained for the first four days in 12:12 LD cycles followed by release into DD. For dose and duration response experiments, cohorts were treated on day 9 with midpoint of anaesthesia imposed at CT4. Using infrared night-vision equipment, cohorts of 36 flies (one DAM monitor full) were placed in an airtight container supplied with either isoflurane titrated in air to the desired concentration, or air alone for the controls. For the phase response curve experiments successive cohorts were given timed pulses of anaesthesia (2% isoflurane) on the fifth day of DD using an Aschoff’s type I protocol. Phase advances and delays were calculated in ClockLab, comparing the regression lines fitted to the offsets of activity before and after the intervention (see actograms Figure S1). A general linear model (GLM) with group (anaesthesia or control) and CT time (in two-hour bins) as factors was used to determine the effects on the phase shifts. Tukey’s HSD post-hoc analysis was used to account for multiple comparisons.

### 4.2. Clock Gene Expression Experiments

We used *period-luciferase* reporter (8.0-*luc*) that only expresses *per*-luciferase fusion gene in subsets of the dorsal clock neurons of the adult brain [2,18] to investigate how GA affects molecular clock oscillations in the central brain. To establish the temporal patterns of *period* gene expression, 8.0-luc flies were placed into 96-well plates in every second well (48 per 96-well plate), containing 15 mM d-luciferin potassium salt, 1% agar, and 5% sucrose media. The level of *per* expression was recorded every 30 min by an EnSpire plate reader housed in an environmental cabinet as described in the behavioural protocol above. Phase responses were measured to anaesthesia (6 h of 2% Isoflurane) conducted on separate cohorts of flies every two hours over the 24 h circadian cycle. Data from flies were included in the analysis if it was continuous, which we defined as “the animals survived the whole experiment and there was daily activity”. Animals were excluded from further analysis if they were arrhythmic, defined in ClockLab as “not exceeding the chi-square test of significance”.

### 4.3. Analysis of Bioluminescence Data

A standard combined cosinor and exponential decay model was fitted to the data to compensate the depletion of the luciferin in the media and to calculate the phase of the rhythm using Equation (1):

*f*(*x*) = *Ae*^−*dt*^ cos (*ωt* − *φ*) + *B* (1)

The combined curve was fitted by regression to the data before and after the anaesthesia intervention from which the acrophases of the cosine curves were calculated. The phase shift was then obtained by the difference between the acrophases relative to the period. The same criteria for data exclusion (death or arrhythmicity) were used as for the activity experiments. We analysed the data using GLM using group and CT as factors and applied post-hoc Tukey HSD for multiple comparisons.

## Supplementary Materials

The following are available online at https://www.mdpi.com/2624-5175/2/4/32/s1, Figure S1: Individual actograms, Figure S2: Individual bioluminescence traces, Table S1: The effect of duration of anaesthesia on phase, Table S2: The effect of concentration of anaesthesia on phase, Table S3: Behavioural phase response, Table S4: PERIOD expression response, Table S5: GLM model of behavioural response, Table S6: GLM model PERIOD expression.

## References

[1] Franks N.P. (2006). Molecular targets underlying general anaesthesia. Br. J. Pharmacol..

[2] Veleri S., Brandes C., Helfrich-Foerster C., Hall J.C., Stanewsky R. (2003). A Self-Sustaining, Light-Entrainable Circadian Oscillator in the Drosophila Brain. Curr. Biol..

[3] Ceriani M.F., HogenEsch J.B., Yanovsky M., Panda S., Straume M., Kay S.A. (2002). Genome-Wide Expression Analysis in DrosophilaReveals Genes Controlling Circadian Behavior. J. Neurosci..

[4] Jaramillo A.M., Zheng X., Zhou Y., Amado D.A., Sheldon A., Sehgal A., Levitan I.B. (2004). Pattern of distribution and cycling of SLOB, Slowpoke channel binding protein, in Drosophila. BMC Neurosci..

[5] Cheeseman J.F., Winnebeck E.C., Millar C.D., Kirkland L.S., Sleigh J., Goodwin M., Pawley M.D.M., Bloch G., Lehmann K., Menzel R. (2012). General anesthesia alters time perception by phase shifting the circadian clock. Proc. Natl. Acad. Sci. USA.

[6] Ko M.L., Jian K., Shi L., Ko G.Y.-P. (2009). Phosphatidylinositol 3 kinase-Akt signaling serves as a circadian output in the retina. J. Neurochem..

[7] Young M.W., Kay S.A. (2001). Time zones: A comparative genetics of circadian clocks. Nat. Rev. Genet..

[8] Lowrey P.L., Takahashi J.S. (2011). Genetics of Circadian Rhythms in Mammalian Model Organisms. BT—The Genetics of Circadian Rhythms. Advances in Genetics.

[9] Meyer-Bernstein E.L., Sehgal A. (2001). Book Review: Molecular Regulation of Circadian Rhythms in Drosophila and Mammals. Neuroscientist.

[10] Stanewsky R. (2002). Clock mechanisms in Drosophila. Cell Tissue Res..

[11] Hardin P.E. (2011). Molecular Genetic Analysis of Circadian Timekeeping in Drosophila BT—The Genetics of Circadian Rhythms. Advances in Genetics.

[12] Aschoff J. (1960). Exogenous and Endogenous Components in Circadian Rhythms. Cold Spring Harb. Symp. Quant. Biol..

[13] Pittendrigh C.S. (1954). On temperature independence in the clock system controlling emergence time in drosophila. Proc. Natl. Acad. Sci. USA.

[14] Nickalls R.W.D., Mapleson W.W. (2003). Age-related iso-MAC charts for isoflurane, sevoflurane and desflurane in man. Br. J. Anaesth..

[15] Nunn M.J.F. (1985). Isoflurane as a routine anaesthetic in general surgical practice. Br. J. Anaesth..

[16] Vanin S., Bhutani S., Montelli S., Menegazzi P., Green E.W., Pegoraro M., Sandrelli F., Costa R., Kyriacou C.P. (2012). Unexpected features of Drosophila circadian behavioural rhythms under natural conditions. Nature.

[17] Rubin E.B., Shemesh Y., Cohen M., Elgavish S., Robertson H.M., Bloch G. (2006). Molecular and phylogenetic analyses reveal mammalian-like clockwork in the honey bee (Apis mellifera) and shed new light on the molecular evolution of the circadian clock. Genome Res..

[18] Yoshii T., Wülbeck C., Sehadova H., Veleri S., Bichler D., Stanewsky R., Helfrich-Förster C., Wulbeck C., Sehadova H., Veleri S. (2009). The Neuropeptide Pigment-Dispersing Factor Adjusts Period and Phase of Drosophila’s Clock. J. Neurosci..

