# Supplementary material for: The Effect of General Anaesthesia on Circadian Rhythms in Behaviour and Clock Gene Expression of Drosophila melanogaster
Source: Clocks Sleep. 2020 Oct 23;2(4):434–41. doi: 10.3390/clockssleep2040032 (PMC7712384; doi:10.3390/clockssleep2040032)
Supplement: Supplementary file 1 [file clockssleep-02-00032-s001.pdf]

Table S1 The effect of duration of anaestheisa on phase

Duration (h)	control	1 h	2 h	3 h	4 h	5 h	6 h
Mean Phase Shift	0.14	0.30	0.22	0.98	0.85	1.10	1.18
SEM	0.10	0.13	0.24	0.20	0.13	0.23	0.16
n	31	30	16	23	27	17	26
p value Tukey-Kramer		0.9853	0.9999	0.0028	0.0133	0.0016	<0.0001

Table S2 The effect of concentration of anaesthesia on phase

Concentration (%)	0%	0.5%	1.0%	1.5%	2.0%	2.50%	3.0%
Mean Phase Shift	0.252	0.581	0.592	1.247	1.455	1.442	1.539
SEM	0.046941378	0.078369495	0.10207113	0.125842721	0.159649518	0.114370328	0.167907413
n	14	15	16	13	13	13	13
p value Tukey-Kramer		0.402	0.3428	<0.0001	<0.0001	<0.0001	<0.0001

Table S3 The behavioural Phase response to a 2% Isoflurane anaesthetic

CT	Mean Phase Shift	SEM	n	Ctrl	SEM	n
0-2	0.85	0.13	30	0.16	0.07	29
2-4	1.25	0.17	42	0.31	0.10	42
4-6	0.78	0.19	40	0.20	0.08	42
6-8	0.57	0.13	42	0.12	0.07	36
8-10	0.17	0.15	54	0.11	0.07	21
10-12	-0.45	0.32	16	0.01	0.09	21
12-14	-0.91	0.22	23	-0.02	0.11	24
14-16	-0.76	0.20	27	-0.13	0.15	20
16-18	-0.53	0.19	16	0.05	0.10	28
18-20	-0.49	0.19	30	-0.17	0.19	20
20-22	-0.11	0.26	26	0.14	0.07	34
22-24	0.26	0.19	37	0.14	0.03	34

Table S4 PERIOD expression phase response to a 2% Isoflurane

CT	Mean Phase Shift	SEM	n	Control	SEM	n
0-2	1.02	0.20	45	0.24	0.07	32
2-4	1.22	0.22	54	0.05	0.06	28
4-6	0.13	0.43	22	-0.02	0.09	22
6-8	-1.68	0.27	34	0.00	0.07	22
8-10	-1.60	0.39	28	-0.01	0.12	7
10-12	-1.55	0.35	19	0.07	0.14	10
12-14	-1.27	0.64	13	-0.33	0.13	11
14-16	-1.84	0.68	12	-0.07	0.12	9
16-18	-1.50	0.32	24	-0.05	0.06	33
18-20	0.13	0.67	20	-0.11	0.07	34
20-22	0.68	0.58	15	-0.14	0.11	14
22-24	1.21	0.38	29	0.32	0.12	11

Table S5 Specific contrasts of the GLM of locomotor behaviour showing effect size and significence levels between CT4 and each other CT.

Dependent Variable: PhaseShift

Tukey HSD

(I) CT	(J) CT	Mean Differenc	Std. Error	Sig.	95% Confidence Interval	
					Lower Bound	Upper Bound
4	2	0.2718	0.14505	0.775	-0.2038	0.7475
	0.2984	0.13256	0.513	-0.1363	0.733	0.733
	0.4218	0.13427	0.075	-0.0185	0.8621	0.8621
	.6302*	0.13566	<0.001	0.1854	1.075	1.075
	.9709*	0.16849	<0.001	0.4184	1.5233	1.5233
	1.2385*	0.15555	<0.001	0.7284	1.7485	1.7485
	1.2742*	0.15555	<0.001	0.7642	1.7843	1.7843
	.9439*	0.15891	<0.001	0.4229	1.465	1.465
	1.1423*	0.15252	<0.001	0.6421	1.6424	1.6424
	.7518*	0.14434	<0.001	0.2785	1.2251	1.2251
	.5788*	0.13766	0.002	0.1275	1.0302	1.0302

Based on observed means.

The error term is Mean Square(Error) = 0.729.

* The mean difference is significant at the 0.05 level.

Table S6 Specific contrasts of the GLM of PERIOD expression showing effect size and significence levels between CT4 and each other CT.

Dependent Variable: PhaseShift

Tukey HSD

(I) CT	(J) CT	Mean Differenc	Std. Error	Sig.	95% Confidence Interval	
					Lower Bound	Upper Bound
4	2	0.1289	0.23228	1	-0.6337	0.8914
	6	0.7658	0.27354	0.183	-0.1322	1.6638
	8	1.8423*	0.25375	<0.001	1.0093	2.6754
	10	2.1084*	0.29554	<0.001	1.1381	3.0786
	12	1.8153*	0.31624	<0.001	0.7771	2.8535
	14	1.6599*	0.33971	<0.001	0.5446	2.7751
	16	1.9025*	0.35799	<0.001	0.7273	3.0778
	18	1.4823*	0.25242	<0.001	0.6536	2.311
	20	.8428*	0.25653	0.05	0.0006	1.6849
	22	0.5366	0.31624	0.869	-0.5016	1.5748
	24	-0.1602	0.28472	1	-1.0949	0.7746

Based on observed means.

The error term is Mean Square(Error) = 2.143.

* The mean difference is significant at the 0.05 level.

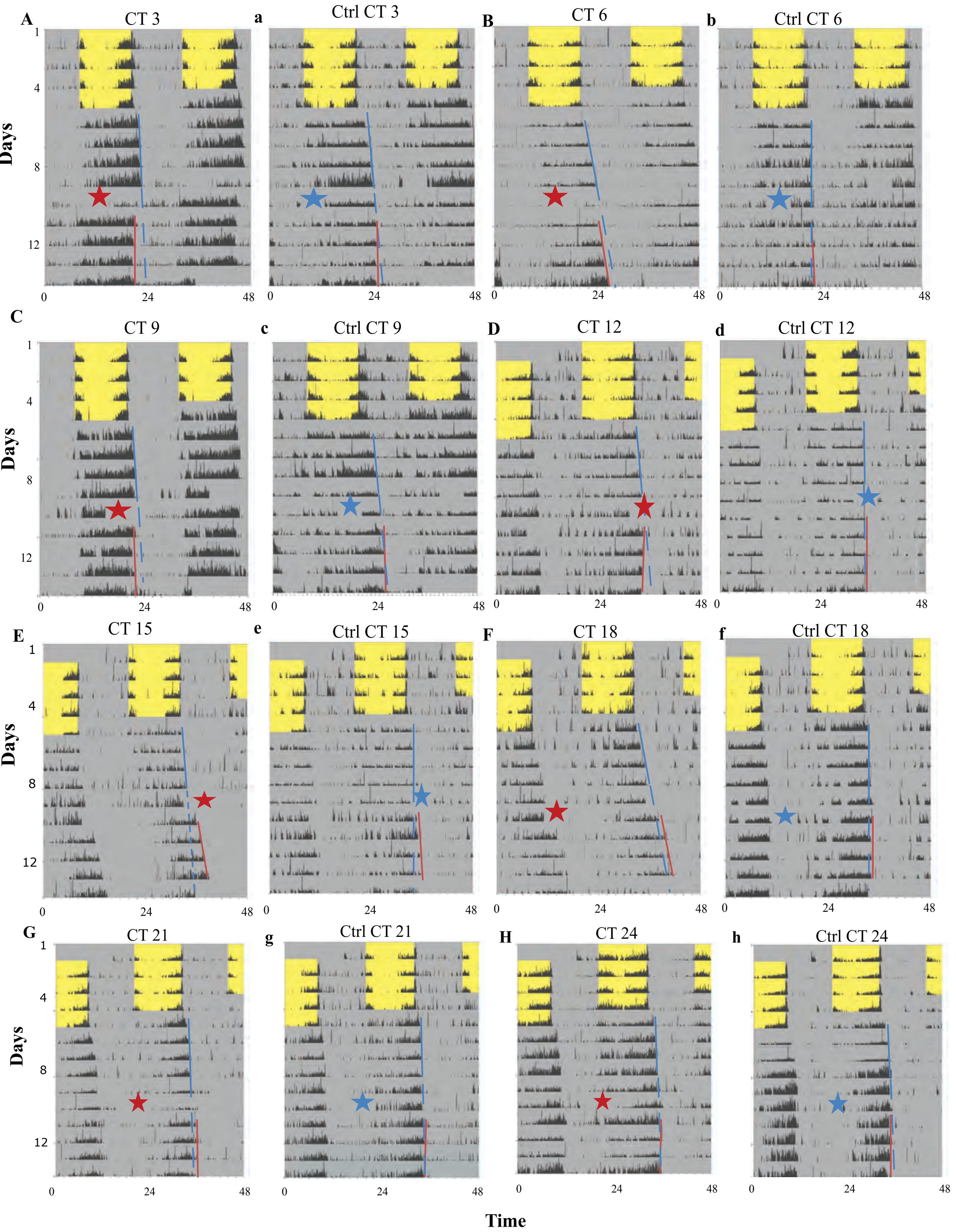

Figure S1. Representative example actograms of individuals showing the effect of anaesthesia administered across the circadian cycle. Here these actograms are shown in three-hour bins rather than the two-hour bins in the main analysis. Uppercase letters denote the anaesthetic group (with red stars denoting time of anaesthesia) and lowercase letters show associated controls (blue stars) at the same CT. Blue lines show regression line fitted to offsets before the intervention. Red lines show regression fit of offsets after intervention.

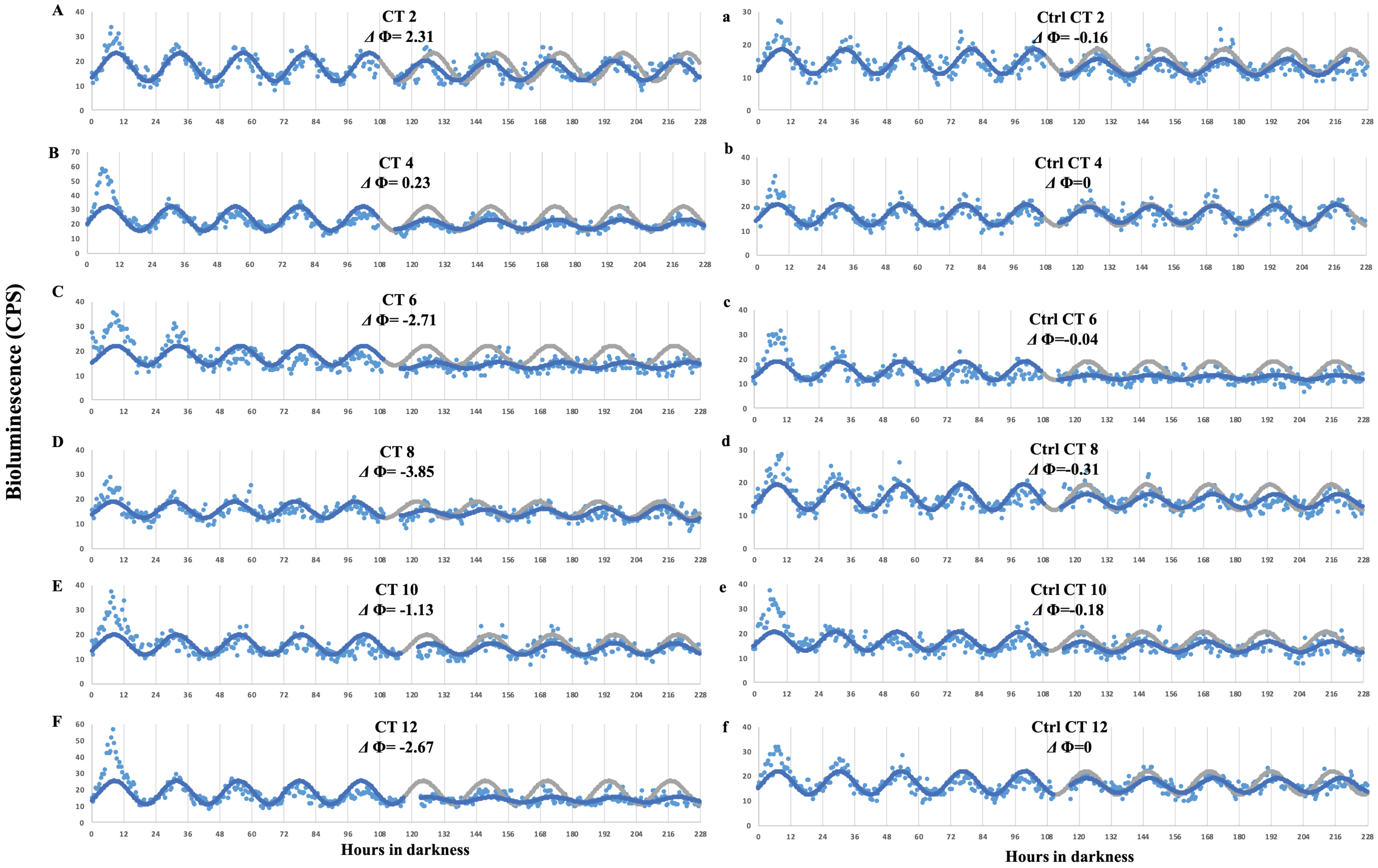

A
CT 2
⊿ Φ= 2.31
a
Ctrl CT 2
⊿ Φ= -0.16
B
CT 4
⊿ Φ= 0.23
b
Ctrl CT 4
⊿ Φ=0
C
CT 6
⊿ Φ= -2.71
c
Ctrl CT 6
⊿ Φ=-0.04
D
CT 8
⊿ Φ= -3.85
d
Ctrl CT 8
⊿ Φ=-0.31
E
CT 10
⊿ Φ= -1.13
e
Ctrl CT 10
⊿ Φ=-0.18
F
CT 12
⊿ Φ= -2.67
f
Ctrl CT 12
⊿ Φ=0
Bioluminescence (CPS)
Hours in darkness
Hours in darkness

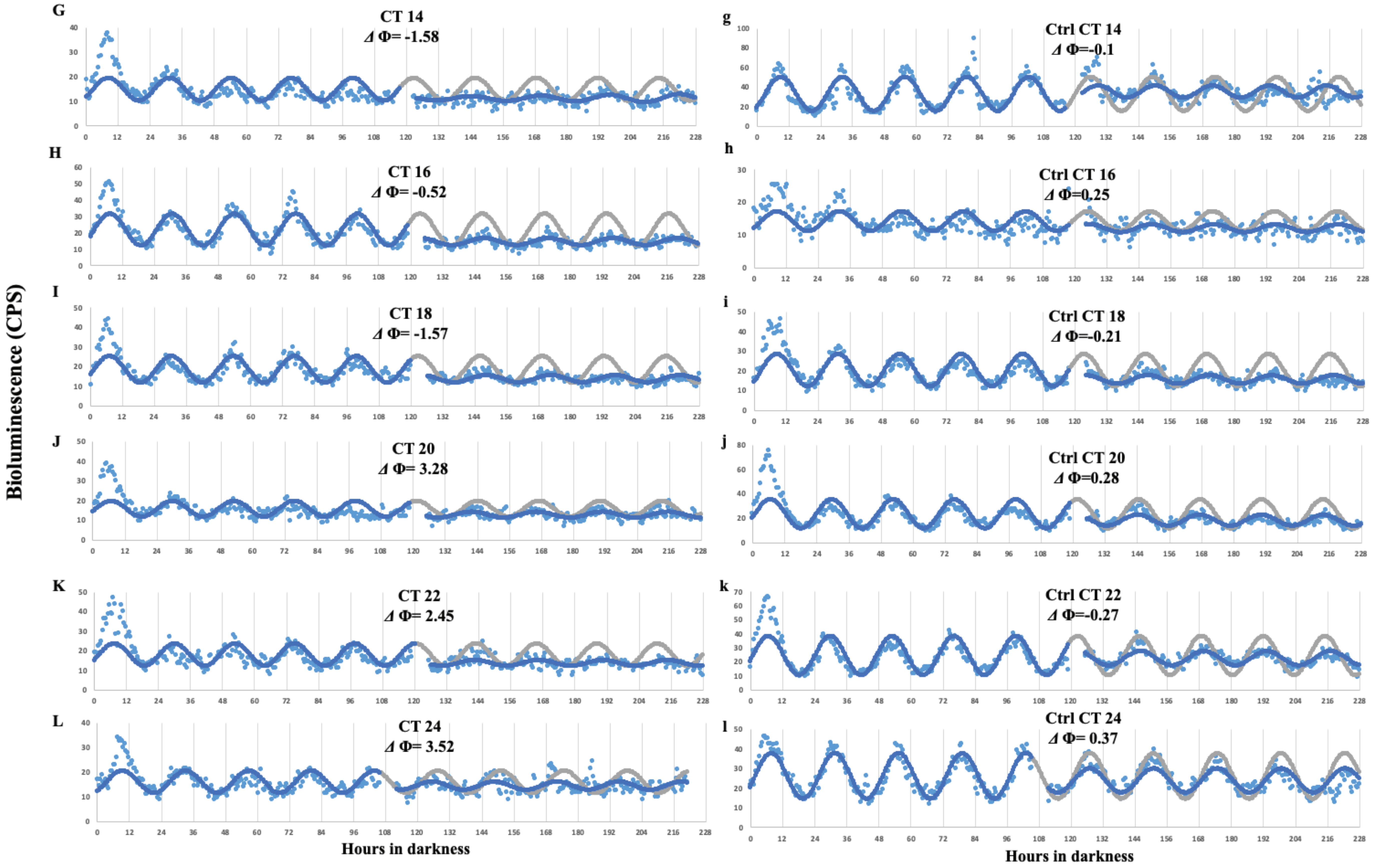

Figure S2. Example bioluminescence traces of individuals across the circadian cycle. Uppercase letters denote Anaesthetic intervention group and associated lower case letters show associated control at the same CT. The best model fits to the data are shown in blue lines (both before and after the intervention). Grey lines show the continuation of the pre-intervention model for comparison. Phase change denoted by ΔΦ.
